# Fissurectomy Versus Lateral Internal Sphincterotomy in the Treatment of Chronic Anal Fissure: A Randomized Control Trial

**DOI:** 10.7759/cureus.18363

**Published:** 2021-09-28

**Authors:** Bipin Kishore Bara, Sujit Kumar Mohanty, Satya Narayan Behera, Ashok Kumar Sahoo, Santanu Kumar Swain

**Affiliations:** 1 Surgery, Santha Bhima Bhoi Medical College and Hospital, Balangir, IND; 2 Surgery, Srirama Chandra Bhanja Medical College and Hospital, Cuttack, IND; 3 Surgery, Jawaharlal Institute of Postgraduate Medical Education & Research, Puducherry, IND

**Keywords:** catheterisation, sitz bath, incontinence, high fibre diet, fissures

## Abstract

Introduction

An anal fissure is defined as a longitudinal split in the distal anoderm which extends from the anal verge to the dentate line. Fissures can be of primary or secondary type. The posterior midline is the most common location for primary fissures, while, anterior primary fissures, though rare, are more common in females. The cause of primary fissure is idiopathic. But secondary fissures are associated with other systemic diseases and can occur at an abnormal position anywhere in the anoderm. A high percentage of acute fissures heal spontaneously within three weeks with conservative medical management comprising of a high fiber diet, warm sitz bath, and topical analgesic with steroids. Secondary anal fissures will not heal in any form of treatment until the primary cause is addressed. These fissures often need surgical treatment. The lateral internal sphincterotomy (LIS) is one of the most practiced treatments for chronic anal fissure. Nonetheless, anal incontinence is one of the worrisome complications of LIS. Fissurectomy is one of the options among those techniques which address the issues with LIS. Some studies showed that patients with chronic fissures who are refractory to medical treatment responded well to fissurectomy. Hence, this study was conducted to compare the outcomes of fissurectomy and lateral internal sphincterotomy in the treatment of chronic anal fissure and compare recurrence and postoperative complications among both the procedures.

Methods

All consecutive patients attending the department of surgery with chronic fissure and age above 18 years were included in the study. All the included patients were randomized into two groups (fissurectomy and LIS) using the serially numbered opaque-sealed envelope (SNOSE) technique. The patients were discharged on the third day. The first visit was scheduled after two weeks and subsequent visits on the first and second months. Then the patients were followed up by telephonic conversation for the next six months. At the end of the follow-up, post-surgical complications were enquired, recorded, and interpreted.

Results

In the present study, out of a total of 87 patients, 80 patients were included in the study. Among all the patients, 16 patients (20%) developed retention of urine. Four patients in the LIS group showed retention of urine whereas in the fissurectomy group it was twelve. The difference was not statistically significant (p-value: 0.025). A total of 10 patients required catheterization postoperatively. More patients in the fissurectomy group developed incontinence to flatus (p-value: 0.02). Incontinence to liquid and solid was significantly higher in the fissurectomy group (p-value: 0.03 and 0.002, respectively).

Conclusion

In the present study, it was found that LIS was a better treatment option for chronic anal fissure than Fissurectomy. The postoperative complications were less in LIS than in fissurectomy. But the recurrence was higher in the LIS group while there was no recurrence in the fissurectomy group.

## Introduction

An anal fissure is a common benign anorectal problem resulting in pain and bleeding during defecation. An anal fissure is defined as a longitudinal split in the distal anoderm which extends from the anal verge to the dentate line [1]. The pain is excruciating, persists for few hours after defecation, and is quite disturbing in day-to-day life. The bleeding occurs as a streak of blood in the stool or sometimes it stains the toilet paper. Fissures can be of primary or secondary type. According to Pelta, primary fissures occur most commonly in the posterior midline (90%) but about 10% can occur in the anterior position, which is more common in females [2]. A study done by Boulos revealed the incidence of the posterior and anterior fissures was 85.7% and 14.2%, respectively [3]. The cause of primary fissure is idiopathic. A few studies hypothesized that a relatively ischemic environment formed by a decrease in blood flow to the midline portion of the anus with associated sphincter spasm may be the reason for these anal fissures [4,5]. The additional stretching of the anal canal causing worsening of the tear is usually prevented by the existing natural anal spasm. A vicious cycle sets in comprising of the anal spasm which exacerbates the ischemia and prevents the healing of the fissure. This, in turn, prevents the further tearing of fissures by sustaining the anal spam. And once the cycle commences, the chance of spontaneous healing of the fissure goes down and the edges of the fissure get more fibrosed, giving rise to a chronic fissure [6]. But secondary fissures are associated with other systemic diseases (like HIV infection, Crohn’s disease, Tuberculosis, diabetes, immunosuppression, anorectal malignancy, etc.) and can occur at an abnormal position anywhere in the anoderm. A high percentage of acute fissures heal spontaneously within three weeks with conservative medical management comprising of a high fiber diet, warm sitz bath, and topical analgesic with steroid [7]. But the same treatment is not fruitful for treating a chronic anal fissure. Secondary anal fissures will not heal in any form of treatment until the primary cause is addressed. Chronic anal fissure overlies the fibers of the internal anal sphincter with a sentinel skin tag externally and internally it extends up to a hypertrophied papilla. These fissures often need surgical treatment. Basing on the principle of lowering the internal anal sphincter tone and avoiding the risk of fecal incontinence, various surgical treatment modalities have been developed in the last two decades. The lateral internal sphincterotomy is one of the most practiced treatments for chronic anal fissure. Nonetheless, it is having its complications like anal incontinence in approximately 30% of the cases [8]. Therefore, a lot of other techniques have been proposed for chronic anal fissure. Fissurectomy is one of the options among those techniques to treat chronic anal fissures. Many surgeons used this technique in patients with a high risk of incontinence, such as old age people, multiparous women, patients with the normal anal tone, and patients with a previous history of anorectal surgery [9]. Some studies showed that patients with chronic fissures who are refractory to medical treatment responded well to fissurectomy [10]. Hence, this study was conducted to compare the outcomes of fissurectomy and lateral internal sphincterotomy (LIS) in the treatment of chronic anal fissure and compare recurrence and postoperative complications among both the procedures.

## Materials and methods

The study was conducted in the department of general surgery at a tertiary care hospital in Eastern India from February 2019 to January 2021. The study was approved by the ethics committee of the institute and has been performed in accordance with the ethical standards laid down in an appropriate version of the Declaration of Helsinki (as revised in Brazil 2013).

All consecutive patients attending the department of surgery with chronic anal fissure and age above 18 years were included in the study. The details of the patients and the findings were recorded. Patients with multiple anal fissures, recurrent fissures, fissures with hemorrhoids and fistula, fissures associated with malignancies, fissures secondary to specific diseases like tuberculosis, etc., pregnant women were excluded from the study.

The study was designed as a prospective, open-labeled, parallel-arm, randomized controlled trial (RCT). Block randomization was carried out using a computer program with randomly selected block sizes of four and six. Allocation concealment was ensured by a serially numbered opaque-sealed envelope (SNOSE).

All consecutive patients with chronic anal fissure were recruited in the primary cohort, after obtaining written informed consent. All of them were initially managed according to the same conservative standard protocol, namely, medical treatment including dietary modification, stool softeners, and local ointments. All patients were assessed five to six weeks after the first visit, and surgical treatment was offered in the case of refractory symptoms. All the included patients were randomized into two groups (fissurectomy and LIS) using the SNOSE technique. The patients were started on stool softener two days before surgery. They were kept on a liquid diet 24 hours before the operation. On the day of operation, the patients were given an enema to avoid any soiling during surgery. All the patients underwent surgery in the lithotomy position after giving spinal anesthesia. Prophylactic parenteral antibiotics were administered just before the procedure according to a standardized protocol.

In the fissurectomy group, anal dilatation was done for four minutes by using a four-finger technique followed by fissurectomy. The fissure was excised using a scalpel, and the wound was curated till a healthy margin was reached up to the level of the internal sphincter. Thus a fresh ulcer was made without scar tissue and was allowed to heal by secondary intention. The presence of any concomitant skin tag or sentinel pile was also excised.

In the LIS group, the surgery was done using the closed method. A bivalve speculum was inserted into the anal canal. The groove between the internal and external anal sphincter was felt with the left-hand index finger. Then a scalpel was inserted into the groove and cautiously turned towards the internal sphincter to divide the muscle partially at the level of the apex of the fissure.

Hemostasis was achieved in both procedures.

Then an anal pack was given to stop any minor bleeding which was taken out after 24 hours and an oral liquid diet was started after four hours. All the patients were kept on IV antibiotics and analgesics for an appropriate period. The patients were discharged on the third day after a final look into the wound.

They were advised to do a warm sitz bath and use a stool softener for two to three weeks along with local ointment to lubricate the anal canal. The first visit was scheduled after two weeks and subsequent visits on the first and second months. Then the patients were followed up by telephonic conversation for the next six months. At the end of the follow-up, post-surgical complications were enquired, recorded, and interpreted.

Statistical analysis

Data were analyzed by using SPSS Statistics for Windows, Version 20.0 (IBM SPSS Statistics for Windows, Version 20.0. Armonk, NY: IBM Corp). Appropriate statistical tests were used to compare the results of fissurectomy and LIS. Descriptive results were expressed as mean and SD of various parameters. p < 0.05 was considered statistically significant.

## Results

**Figure 1:**
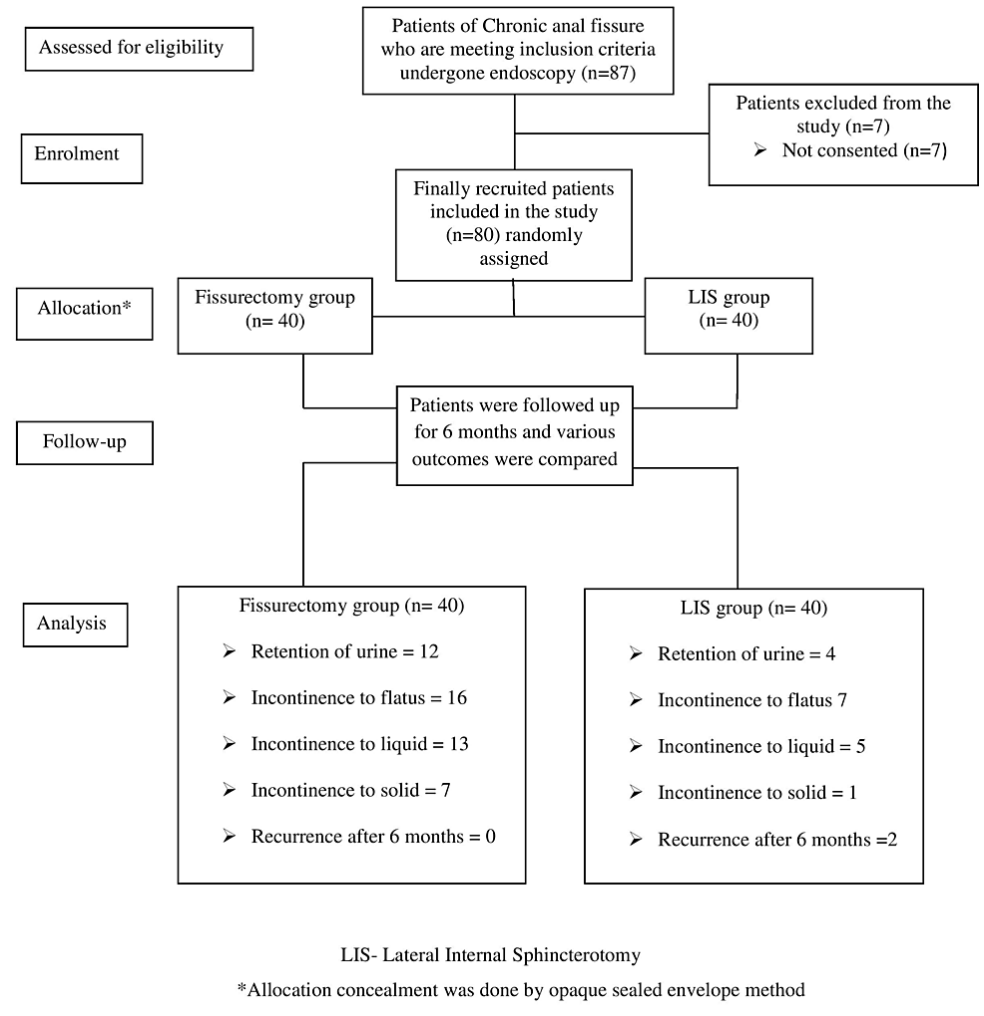
The overall scheme as per CONSORT flowchart. CONSORT: CONsolidated Standards Of Reporting Trials.

In the present study, out of a total of 87 patients, 80 patients were included in the study (Figure 1).

All the seven patients who were excluded from the study had not given consent for the same.

The mean age in fissurectomy and LIS groups was 37.23 ± 11.231 and 39.342 ± 10.246 years, respectively.

There were 19 males (47.5 %) and 21 females (52.5%) in the fissurectomy arm while the LIS arm comprised 18 males (45%) and 22 females (55%). The gender distribution between the groups was also comparable and the male-to-female ratio in both the groups did not vary significantly (48/52% vs. 45/55%; p = 0.714). After surgery, almost all patients got relief from pain and bleeding within one week period.

The postoperative complications were depicted in Table 1 and Table 2 for the fissurectomy group and LIS group, respectively.

**Table 1:** Complications in fissurectomy group.

	Complications	Male (n=20)	Female (n=20)	Total (n=40)
1	Retention of urine	7	5	12
2	Incontinence to flatus	6	10	16
3	Incontinence to liquid	4	9	13
4	Incontinence to solid	2	5	7
5	Recurrence after 6 months	0	0	0

**Table 2:** Complications in lateral internal sphincterotomy (LIS) group.

	Complications	Male (n=20)	Female (n=20)	Total (n=40)
1	Retention of urine	2	2	4
2	Incontinence to flatus	1	6	7
3	Incontinence to liquid	1	4	5
4	Incontinence to solid	0	1	1
5	Recurrence after 6 months	2	0	2

In the fissurectomy group, 12 patients (30%) developed retention of urine, of whom seven were male and the rest were female. A total of 16 patients (40%) showed incontinence to flatus which was found to be more in females (six males and 10 females). Thirteen patients became incontinent to liquid (32.5%), most of whom were female. Incontinence to solid was seen in seven patients (17.5%), with five of them being female. There was no recurrence in this group after six months of follow-up.

In the LIS group, four patients (10%) developed retention of urine, two each male and female. A total of seven patients (17.5%) showed incontinence to flatus which was found to be more in females (one male and six females). Five patients became incontinent to liquid (12.5%), most of whom were female. Incontinence to solid was seen in one patient (2.5%), who was a female. A total of two male patients (5%) had recurrence after six months.

Table 3 illustrated the comparison of postoperative complications among both groups.

**Table 3:** Comparison of complications between fissurectomy and lateral internal sphincterotomy (LIS) group.

	Complications	Fissurectomy group	LIS group	p-value
1	Retention of urine	12	4	0.025
2	Incontinence to flatus	16	7	0.02
3	Incontinence to liquid	13	5	0.03
4	Incontinence to solid	7	1	0.02

Among all the patients, 16 patients (20%) developed retention of urine. Four patients in the LIS group showed retention of urine whereas in the fissurectomy group it was 12. The difference was not statistically significant (p-value: 0.025). A total of 10 patients required catheterization postoperatively. More patients in the fissurectomy group developed incontinence to flatus (p-value: 0.02). Incontinence to liquid and solid was significantly higher in the fissurectomy group (p-value: 0.03 and 0.002, respectively).

None of the patients developed key-hole deformity in both groups.

## Discussion

Sphincterotomy as the surgical treatment of choice for chronic anal fissure was first described by Boyer [11]. Following that, a lot of procedures have been developed to address the issue. Fissurectomy, anal dilatation, posterior and lateral sphincterotomy, and advancement flap are few proposed procedures among them [12]. In lateral internal sphincterotomy, the internal sphincter is divided in its distal third away from the fissure itself - either in the right or left lateral position [13]. The main aim of LIS is to increase the blood flow of the anoderm by decreasing the maximum anal sphincter pressure by 18%-50%. The rate of healing of fissure was ranging from 93% to 95% in the open technique which scores to 90% to 97% by using the close technique. In the present study, the close technique was carried out. Still, fissurectomy with manual dilatation is advocated in young adults with very high sphincter tone. In France, the procedure of choice for chronic anal fissure, refractory to medical management, is fissurectomy. This is based on the anecdotal and published evidence that fissurectomy exhibits a comparable rate of healing as compared to LIS. A German study demonstrated favorable results with fissurectomy as the procedure of choice for chronic anal fissure [14]. In the present study, after surgery, almost all patients got relief from pain and bleeding within one week period.

Up to 35% of patients developed complications following LIS in a study done by Khubchadani et al [15]. Another study conducted by Littlejohn et al documented 35% of patients complained of minor straining following LIS [16]. The risk factors for continence disturbance include age over 40 years, female sex, history of vaginal delivery, anterior fissure, the addition of synchronous anorectal procedure, and technique [17]. Charua et al. found that 6.5% of patients following LIS developed minimal fecal incontinence at three months post-surgery [18]. As per the study done by Nyam et al., the patients developing incontinence to flatus, mild soiling, and gross incontinence were 31%, 39%, and 23%, respectively [19].

A study done by Hoffman and Goliger revealed that patients undergoing LIS had occasional incontinence to flatus and feces [20]. Garcia et al demonstrated that incontinence among patients undergoing LIS was raging from 16.1% to 26.7% [21].

But contrary to the above studies, Walker et al found different results in their study [22]. They conducted a long-term follow-up for 4.3 years in 100 patients who had undergone LIS and found a moderate degree of soiling ranging from 3% to 5 %. Mousavi reported no incidence of fecal soiling and incontinence to flatus [23]. No long-term complication was observed by Adriano Tocchi in his study on LIS [24].

Aziz in his study on 146 patients found a cure rate of 97.5% over three months period and there was no long-term complication and patient compliance was 100% [25]. Flatus incontinence was found in 4.1% of patients but that was transitory. Schouten WR et al. in his study on LIS found pain relief in 98% of cases [26]. Daniel O in his study of chronic anal fissures found that LIS is an effective procedure with a high rate of resolution of symptoms but with a risk of temporary or permanent incontinence [27].

In the present study, four patients in the LIS group showed retention of urine whereas in the fissurectomy group it was twelve. Among them, 10 patients required catheterization postoperatively. More patients in the fissurectomy group developed incontinence to flatus (p-value: 0.02). Incontinence to liquid and solid was significantly higher in the fissurectomy group (p-value: 0.03 and 0.002, respectively). No recurrence was found in the fissurectomy group. Unfortunately, two male patients developed recurrence after undergoing LIS.

As the anal canal is shorter in females, the risk of sphincter injury is more during delivery [28]. According to several studies, the LIS procedure becomes more extensive in females than in males [29,30]. This may be one of the reasons for getting more complications in female patients in the present study.

Keyhole deformity is one of the worst complications of fissurectomy which may lead to fecal soiling. Fortunately, in the present study, none of the patients developed such complications.

There are few limitations in the present study. The sample size of the study was less. It was a single-center study.

## Conclusions

In the present study, it was found that LIS was a better treatment option for chronic anal fissure than fissurectomy. The postoperative complications were less in LIS than in fissurectomy. But the recurrence was higher in the LIS group while there was no recurrence in the fissurectomy group. Four finger dilatation followed by fissurectomy is a better option for young male patients whose resting anal sphincter pressure is high, and also in females with previous obstetric trauma, and short anal canal. As the sample size is small, further studies are needed to establish the conclusion.
